# Recombinant Zika Virus Subunits Are Immunogenic and Efficacious in Mice

**DOI:** 10.1128/mSphere.00576-17

**Published:** 2018-01-10

**Authors:** Albert To, Liana O. Medina, Kenji O. Mfuh, Michael M. Lieberman, Teri Ann S. Wong, Madhuri Namekar, Eileen Nakano, Chih-Yun Lai, Mukesh Kumar, Vivek R. Nerurkar, Axel T. Lehrer

**Affiliations:** a Department of Tropical Medicine, Medical Microbiology, and Pharmacology, John A. Burns School of Medicine, University of Hawaii, Honolulu, Hawaii, USA; b Pacific Center for Emerging Infectious Diseases Research, John A. Burns School of Medicine, University of Hawaii, Honolulu, Hawaii, USA; University of Maryland School of Medicine

**Keywords:** recombinant, subunit, vaccine, Zika virus

## Abstract

Following the 2015 Zika virus (ZIKV) outbreaks in the South Pacific, Caribbean, and Americas, ZIKV has emerged as a serious threat due to its association with infantile microcephaly and other neurologic disorders. Despite an international effort to develop a safe and effective vaccine to combat congenital Zika syndrome and ZIKV infection, only DNA and mRNA vaccines encoding the precursor membrane (prM) and envelope (E) proteins, an inactivated-ZIKV vaccine, and a measles virus-based ZIKV vaccine are currently in phase I or II (prM/E DNA) clinical trials. A ZIKV vaccine based on a nonreplicating, recombinant subunit platform offers a higher safety profile than other ZIKV vaccine candidates but is still highly immunogenic, inducing high virus-neutralizing antibody titers. Here, we describe the production and purification of *Drosophila melanogaster* S2 insect cell-derived, soluble ZIKV E protein and evaluate its immunogenicity and efficacy in three different mouse strains. As expected, significant virus-specific antibody titers were observed when using formulations containing clinically relevant adjuvants. Immunized mice challenged with live virus demonstrate inhibition of virus replication. Importantly, plaque reduction neutralization tests (PRNTs) indicate the high-titer production of neutralizing antibodies, a correlate of protection in the defense against ZIKV infection. ZIKV challenge of immunocompetent mice led to full protection against viremia with two doses of adjuvanted vaccine candidates. These data demonstrate a proof of concept and establish recombinant subunit immunogens as an effective vaccine candidate against ZIKV infection.

**IMPORTANCE** The recent outbreaks of Zika virus (ZIKV) infection in French Polynesia, the Caribbean, and the Americas have highlighted the severe neuropathological sequelae that such an infection may cause. The development of a safe, effective ZIKV vaccine is critical for several reasons: (i) the difficulty in diagnosing an active infection due to common nonspecific symptoms, (ii) the lack of a specific antiviral therapy, and (iii) the potentially devastating pathological effects of *in utero* infection. Moreover, a vaccine with an excellent safety profile, such as a nonreplicating, noninfectious vaccine, would be ideal for high-risk people (e.g., pregnant women, immunocompromised patients, and elderly individuals). This report describes the development of a recombinant subunit protein vaccine candidate derived from stably transformed insect cells expressing the ZIKV envelope protein *in vitro*, the primary antigen to which effective virus-neutralizing antibodies are engendered by immunized animals for several other flaviviruses; the vaccine candidate elicits effective virus-neutralizing antibodies against ZIKV and provides protection against ZIKV infection in mice.

## Abstract (precis)

The recent outbreaks of Zika virus (ZIKV) infection in French Polynesia, the Caribbean, and the Americas have highlighted the severe neuropathological sequelae that such an infection may cause. The development of a safe, effective ZIKV vaccine is critical for several reasons: (i) the difficulty in diagnosing an active infection due to common nonspecific symptoms, (ii) the lack of a specific antiviral therapy, and (iii) the potentially devastating pathological effects of *in utero* infection. Moreover, a vaccine with an excellent safety profile, such as a nonreplicating, noninfectious vaccine, would be ideal for high-risk people (e.g., pregnant women, immunocompromised patients, and elderly individuals). This report describes the development of a recombinant subunit protein vaccine candidate derived from stably transformed insect cells expressing the ZIKV envelope protein *in vitro*, the primary antigen to which effective virus-neutralizing antibodies are engendered by immunized animals for several other flaviviruses; the vaccine candidate elicits effective virus-neutralizing antibodies against ZIKV and provides protection against ZIKV infection in mice.

## INTRODUCTION

In 1947, Zika virus (ZIKV) was initially isolated from a febrile rhesus macaque in Uganda, followed by its isolation in *Aedes africanus* mosquitoes in 1948 (1). Serological evidence showed human exposure to the virus (2, 3), but no human disease associated with infection was reported until 1954, when symptomatic infection was reported in three patients in Nigeria (4). No major human outbreaks were evident until 2007, when an illness causing rash, conjunctivitis, and arthralgia was observed on Yap Island, Federated States of Micronesia. ZIKV was identified as the causative agent, and an estimated 5,000 people were infected (5). In 2013, a large outbreak was reported in French Polynesia, resulting in 19,000 suspected infections. An increase in Guillain-Barré syndrome concurrent with the outbreak suggested a causal link between the two, and this was the first time that such an association was noted (6, 7). This was followed by a large outbreak in Brazil in 2015, which was the first incidence in the Western Hemisphere (8, 9).

During the Brazilian outbreak in 2015, an increased incidence of newborns with microcephaly was observed, and examination of microcephalic cases showed the presence of virus in infants shortly after birth, indicating transmission of the virus *in utero* (10, 11). Further research found a strong association between maternal ZIKV infection during the first trimester of pregnancy and the risk of microcephaly during the Brazilian outbreak (12), although the current data suggest infection during any trimester of pregnancy may result in ZIKV-associated birth defects (13). Experimental models using mice have shown that ZIKV infection during pregnancy can transmit virus to the fetus through the placenta and cause intrauterine growth restrictions that include cortical deformations (14). The virus has also been shown to infect neural progenitor cells in mice, leading to apoptosis (15, 16). In human cell models that mimic first-trimester brain development *in vitro*, ZIKV infection has been shown to cause cell death in neural stem cells and to reduce the growth of brain organoids, as well as to disrupt the development of neurospheres (17).

Currently, there are no commercially available vaccines against ZIKV, but vaccine candidates from a variety of different platforms are being explored. A purified, inactivated ZIKV based on the Puerto Rican ZIKV strain has been successfully demonstrated to protect against challenge with both homologous and nonhomologous strains in murine and nonhuman primate (NHP) models (18). The DNA vaccine platform using the ZIKV precursor membrane and envelope (prM-E) genes has also been tested in mice and NHPs (18–20). One vaccine candidate uses constructs from the prM-E gene sequences of the French Polynesian strain to create subviral particles (20). Parts of the ZIKV prM or E gene were replaced with analogous portions of Japanese encephalitis virus (JEV) to improve expression and secretion (19, 21). Immunization of C57BL/6J and BALB/c mice with VRC5283, containing the JEV prM signal sequence, and VRC5288, a modified VRC5283 strain with a JEV C-terminus substitution, yields ZIKV-specific neutralizing antibodies (19). Other DNA vaccines have used the prM-E sequences from the Brazilian strain and have shown protection against both homologous and nonhomologous ZIKV strains in BALB/c, SJL, and C57BL/6J mice after two doses (20). A third vaccine platform using a rhesus adenovirus vector has been shown to elicit antibody responses against a wide array of ZIKV E protein epitopes and protect against challenge with a homologous strain (22). There are several ongoing clinical trials that use either a purified, inactivated-ZIKV vaccine (ZPIV), a modified mRNA, a measles virus-based ZIKV vaccine (MV-ZIKA), or a DNA vaccine platform.

In this study, we describe the production of a novel recombinant subunit vaccine candidate comprised of the ZIKV envelope protein (E) ectodomain and two clinically relevant adjuvants and evaluate its immunogenicity in Swiss Webster (SW), BALB/c, and C57BL/6 mice. We also assess efficacy against intravenous ZIKV challenge in immunocompetent, adult BALB/c mice as a ZIKV infection model (20). Vaccines based on this platform have been developed previously against other flaviviruses, e.g., dengue virus (DENV) (24) and West Nile virus (WNV) (25), and have been shown to have excellent safety profiles, robust immunogenicity, and high protective efficacy (24–28).

## RESULTS

### Expression and purification of ZIKV E protein.

**FIG 1:**
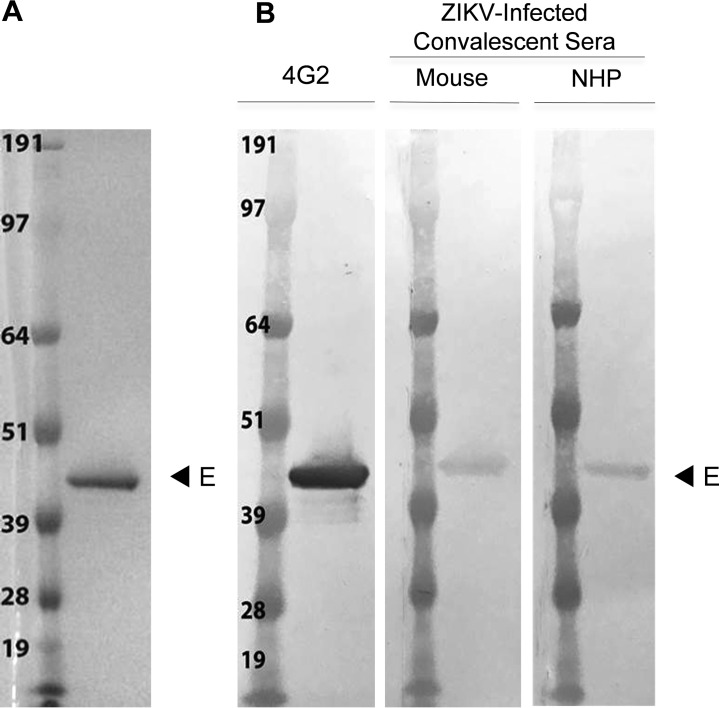
Recombinant ZIKV E protein expressed in *Drosophila* S2 cells after purification using 4G2 MAb. (A) Coomassie blue-stained SDS PAGE gel (4 to 12% polyacrylamide) featuring ZIKV E protein. The molecular weights of the standard marker sizes are shown in kilodaltons (kDa). Purified ZIKV E of approximately 1 μg migrated as a single band. (B) Western blot of purified ZIKV E protein loaded at the same concentration as in panel A. Replicate Western membranes were probed with 4G2 MAb, convalescent ZIKV-infected mouse, or NHP sera.

The supernatant from stably transformed S2 cell lines yielded approximately 5 mg/liter of protein using the WAVE bioreactor. Subsequent purification from the S2 supernatant by immunoaffinity chromatography (IAC), using the anti-flavivirus E protein antibody 4G2, isolated a soluble protein of approximately 45 kDa and indicated a purity of >90% homogeneity, as shown by SDS-PAGE (Fig. 1A). Recognition by the monoclonal antibody (MAb) 4G2 indicated successful purification of the recombinant protein (Fig. 1B, lane 1). To gauge the immunoreactivity of ZIKV E protein, the membrane was probed with convalescent-phase sera from PRVABC59 ZIKV-infected mice and NHPs (Fig. 1B, lanes 2 to 3). Purified ZIKV E protein was detected by both mouse and NHP sera.

### Recombinant ZIKV E antigen is immunogenic in mice.

**FIG 2:**
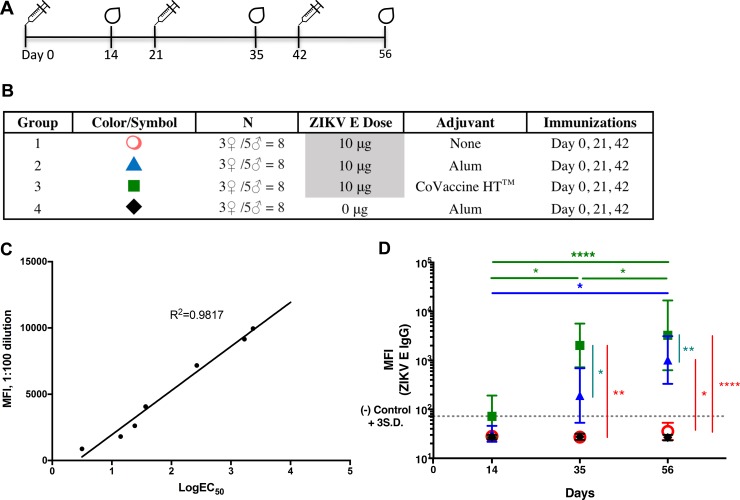
Immunogenicity of recombinant ZIKV E protein in SW mice. (A) Vaccination schedule. The drawings of drops at days 14, 35, and 56 indicate blood draws. (B) Experimental groups. Alum, Imject alum. (C) Median fluorescence intensity (MFI) correlation of EC_50_s with single-point MFIs. The log EC_50_ values of seven different sera were determined by MIA. These values were plotted against the MFI values obtained from these sera at a dilution of 1:100 for each serum sample. (D) Geometric mean titers (GMTs) with 95% confidence intervals (CI) of ZIKV E-specific IgG MFI of sera from all groups of mice immunized with each formulation. S.D., standard deviations. *, *P* < 0.05; **, *P* < 0.01; ****, *P* < 0.0001.

The immunogenicity of purified ZIKV E protein was initially tested in Swiss Webster (SW) mice by immunizing four groups of mice with either three doses (10 μg per dose) of ZIKV E alone, ZIKV E in formulations with the adjuvant aluminum hydroxide (2% Alhydrogel adjuvant or Imject alum, both of which are referred to as alum hereinafter) or CoVaccine HT, or alum alone as a control (Fig. 2A and B). Serum anti-ZIKV E IgG titers were measured by microsphere immunoassay (MIA). We first assessed whether the value of median fluorescence intensity (MFI) at a single dilution in the MIA correlated with the IgG antibody titers calculated as 50% effective concentrations (EC_50_s). Linear regression between the MFI at a 1:100 serum dilution and the EC_50_s of samples with different IgG titers showed good correlation (*R*^2^ = 0.9817) (Fig. 2C), allowing single-point MFI values to be used as an accurate measurement of IgG titers. Therefore, IgG antibody titers are shown as MFIs at the 1:100 dilution for all subsequent assays.

Serum was collected 2 weeks after each immunization and was evaluated for ZIKV E-specific IgG titers. After three doses of the vaccine, mice receiving ZIKV E alone failed to generate a significant humoral response, indicating the need for coadministration with an adjuvant (Fig. 2D). The IgG MFI against ZIKV E protein in mice given the formulation with CoVaccine HT was not significantly above the assay cutoff 2 weeks after the first immunization but was significantly elevated following the second dose and remained high after the third dose. Only a slight increase in titer over that after the second dose was seen after the third immunization. The formulation with alum appeared to yield increased antibody titers after each dose of vaccine; however, the differences in titers after each subsequent vaccine dose were not statistically significant. Only the difference between titers following the first dose and the third dose was significant. These results suggest that two doses of the ZIKV E protein with adjuvant, especially with CoVaccine HT, may be sufficient for eliciting an optimal humoral response.

### Recombinant ZIKV E protein elicits antibody responses in various strains of mice.

**FIG 3:**
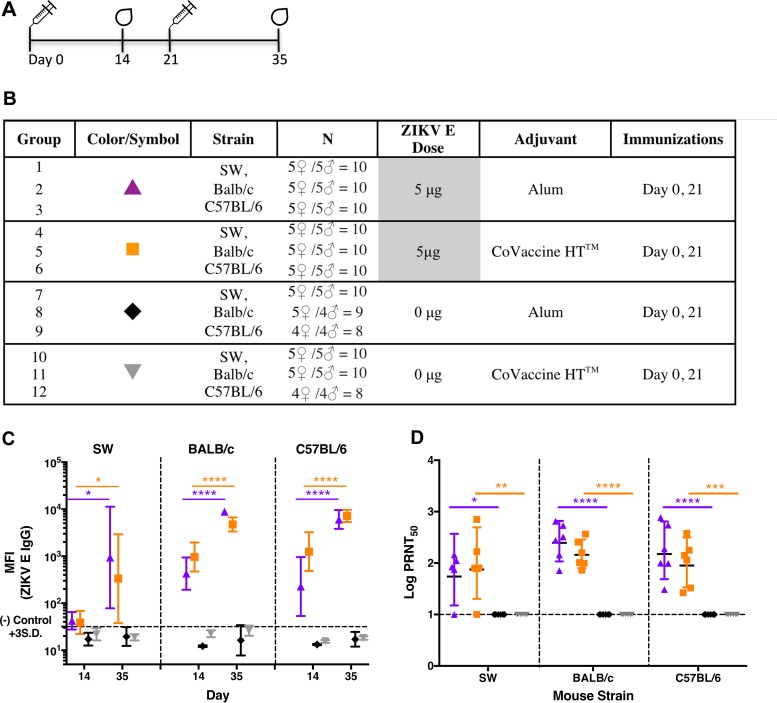
Antibody responses to recombinant ZIKV E protein in three strains of mice. (A) Vaccination schedule. (B) Experimental groups. Alum, 2% Alhydrogel adjuvant. (C) ZIKV E-specific IgG antibodies in the sera from three strains of mice were measured after each immunization using MIA and are plotted as GMTs with 95% CI of total IgG titers. S.D., standard deviations. (D) The neutralizing titers in mouse sera at 2 weeks after the second immunization were measured by PRNT and plotted as the PRNT_50_, the calculated dilution of serum that gave 50% inhibition. Statistical analysis was conducted using unpaired *t* tests and Prism (GraphPad) software. PRNT_50_ values were generated using a fixed, or variable-slope, sigmoidal dose-response model. *, *P* < 0.05; **, *P* < 0.01; ***, *P* < 0.001; ****, *P* < 0.0001.

To characterize the antibody responses generated by the recombinant subunit ZIKV E vaccine candidate in mice with different immunological backgrounds, SW (outbred), BALB/c (Th2-dominant), and C57BL/6 (Th1-dominant) mice were immunized with two doses of 5 μg ZIKV E formulated with either alum or CoVaccine HT at 3-week intervals (Fig. 3A and B). The dose was reduced to 5 μg of E protein in order to determine whether immunogenicity can be sustained with minimal antigen and to accentuate the differences between mouse strains that might otherwise be masked at higher doses. IgG antibody titers in mouse sera were measured 2 weeks following each immunization. Compared to control mice immunized with adjuvant alone, both BALB/c and C57BL/6 mice developed robust humoral responses after the first and second immunization with the recombinant subunit ZIKV E candidate vaccines. No difference in the IgG antibody titers was observed between these two mouse strains (Fig. 3C). An approximate 5-fold increase in IgG titers after the second immunization was seen in both BALB/c and C57BL/6 mice given the CoVaccine HT-adjuvanted vaccine, while the increase was about 20- to 26-fold in mice administered the alum-adjuvanted vaccine. This suggests that CoVaccine HT elicits a stronger immune response to the antigen than alum upon the initial dose but that after the second dose, the alum-adjuvanted formulation matches the titers observed in groups given CoVaccine HT. Although the IgG MFI values obtained from SW mouse antisera were lower than the values obtained from BALB/c and C57BL/6 mice (Fig. 3C), particularly after dose 1, the titers in sera after dose 1 and dose 2 increased nearly 9-fold and about 22-fold for the CoVaccine HT and alum formulations, respectively.

The presence of neutralizing antibodies was assessed after dose 2 by a plaque reduction neutralization test (PRNT) using Vero cells. The results revealed that regardless of their immunological backgrounds, the majority of mice receiving the adjuvanted ZIKV E protein generated good neutralizing antibody titers. Although the serum dilutions yielding 50% virus neutralization (PRNT_50_ titers) were somewhat lower in SW mice than in BALB/c or C57BL/6 mice with both adjuvants, all candidate vaccine groups generated neutralizing antibody titers statistically higher than those in their controls receiving the same adjuvant, suggesting broad efficacy and protective potential (Fig. 3D). Both CoVaccine HT and alum formulations generated comparable titers of neutralizing antibodies.

### Protective efficacy of ZIKV E vaccination in BALB/c mice.

**FIG 4:**
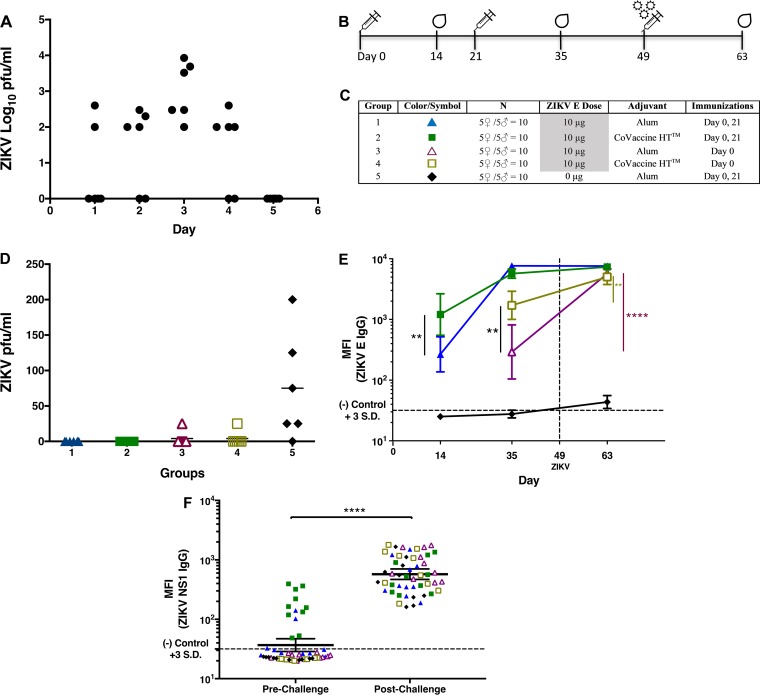
Immunization of mice with recombinant ZIKV E protein vaccine inhibits viral replication postchallenge. (A) Kinetics of viremia in naive BALB/c mice challenged intravenously (i.v.) with 100 PFU of ZIKV Puerto Rican strain PRVABC59. Six animals of both sexes were euthanized daily after infection, and total serum was collected from each animal. Viremia was measured for each serum sample using a standard plaque assay on Vero cells. The limit of detection was 10^1.7^ PFU/ml. (B) Vaccination schedule for the BALB/c mouse challenge study. (C) Experimental groups. Alum, 2% Alhydrogel adjuvant. (D) Viremia in vaccinated and control mice on day 3 after infection. Mice vaccinated once or twice were challenged as described for panel A. Viremia on day 3 postchallenge was measured as described for panel A. The limit of detection was 25 PFU/ml. (E) GMTs expressed as the MFIs (with 95% CI) of ZIKV E-specific IgG titers observed in serum samples collected 2 weeks after immunization and 2 weeks after ZIKV challenge measured by MIA. (F) Anti-NS1 antibody titers in vaccinated and control mice pre- and postchallenge. Antibody titers were measured by MIA on individual serum samples at a 1:100 dilution. **, *P* < 0.01; ****, *P* < 0.0001.

A viremia model of immunocompetent, adult mice was used to assess the protective efficacy provided by immunization with the E protein vaccine candidate formulation. Since BALB/c mice may be resistant to peripheral ZIKV infection (29), mice were inoculated systemically with 100 PFU of ZIKV intravenously (i.v.; through the tail vein) without immunosuppressants. This model had been used previously by Larocca et al. (20) to assess Zika vaccine efficacy. Viremia was determined by plaque assay daily for 5 days after infection. All inoculated animals showed detectable viremia on day 3, which established immunocompetent BALB/c mice as a permissive ZIKV challenge model (Fig. 4A) useful for further experiments.

To examine the protective potential in this challenge model, 4- to 6-week-old BALB/c mice were immunized with one or two doses of 10 μg ZIKV E protein with either CoVaccine HT or alum and challenged 4 weeks after the last vaccination, as depicted in Fig. 4B and C. The presence of viremia was determined by plaque assay on Vero cells using sera collected on the third day after ZIKV challenge by cardiac puncture. This experiment indicated full protection (the absence of viremia) in all 6 mice receiving two 10-μg doses of ZIKV E with either adjuvant (Fig. 4D). Groups receiving a single immunization showed breakthrough viremia in one mouse per group, while the mice in the control group demonstrated viremia in five of the six mice (*P* < 0.01 compared to twice-immunized groups).

To provide additional support for the protective efficacy of the ZIKV E protein vaccine candidate, in a separate experiment, the antibody response postchallenge in vaccinated mice was assessed to determine whether an anamnestic response (4-fold increase in IgG titers) would be observed. The lack of such a response has been regarded as evidence for the inhibition of viral replication postchallenge, indicative of effective protection, which has recently also been used as a secondary outcome measure in various clinical dengue virus vaccine studies (for example, in a recently completed study by Durbin et al. [ClinicalTrials registration no. NCT02317900]). A single dose of ZIKV E protein with CoVaccine HT generated a greater antibody response than the antigen formulation with alum (Fig. 4E), consistent with the study results shown in Fig. 3C.

Two immunizations of ZIKV E with either adjuvant yielded higher IgG titers than one immunization. Although the CoVaccine HT-adjuvanted vaccine induced higher IgG antibody titers after the first immunization, the magnitude of increase in serum IgG titers between the first and second immunizations was higher in mice that received alum-adjuvanted vaccine (26-fold increase) than in mice receiving the CoVaccine HT-adjuvanted vaccine (5-fold increase), consistent with previous results also shown in Fig. 3C. After the second dose, both formulations elicited comparable IgG antibody titers.

To evaluate the humoral response after ZIKV challenge as an indirect marker of protection from viremia, mice were infected i.v. with 100 PFU of ZIKV via tail vein injection, without immunosuppressants, 4 weeks after the last immunization, and sera from all groups were collected 2 weeks postchallenge. In mice immunized with a single dose of the antigen formulation, ZIKV challenge resulted in an approximately 19-fold increase in IgG MFIs when mice were immunized with ZIKV E and alum and in a 3-fold increase when mice were immunized with the antigen plus CoVaccine HT compared to prechallenge titers, effectively demonstrating an anamnestic response in these animals (Fig. 4E). In contrast, no significant difference in post- versus prechallenge IgG titers was observed in mice receiving two doses of either candidate vaccine formulation. The lack of an anamnestic response in these animals suggests that two vaccinations successfully prevented systemic viral replication.

To confirm the successful infection of all mice, IgG titers against ZIKV NS1 were also measured pre- and postchallenge using an in-house-developed anti-ZIKV NS1 IgG MIA. A marked increase in anti-NS1 IgG titers (as MFI) postchallenge (Fig. 4F) suggests that all mice were productively infected with the ZIKV. The MFIs against NS1 were below the cutoff for most of the mice prechallenge, but a few mice, mainly from the CoVaccine HT-containing group, showed low reactivity, possibly due to an immune response against a low level of common S2-derived proteins carried through purification of both E and NS1.

### Sera from ZIKV E-immunized mice protect against ZIKV challenge.

**FIG 5:**
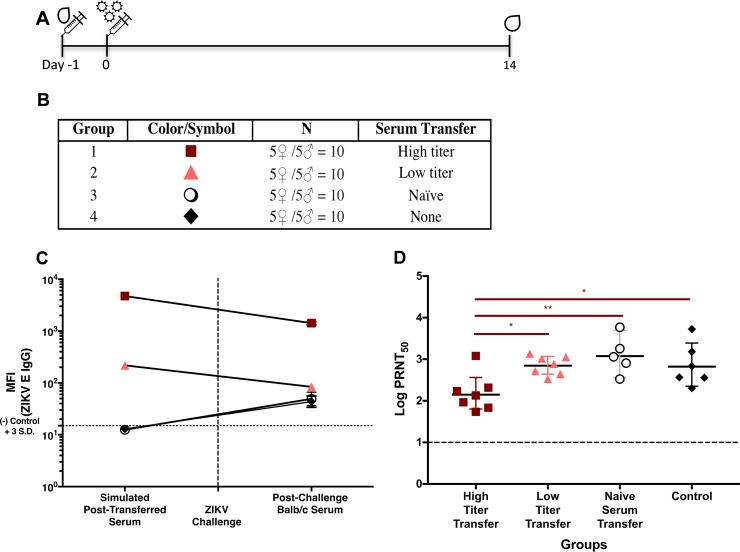
Passive protection of mice with antiserum to ZIKV E protein. (A) Serum transfer and challenge schedule for the BALB/c passive-protection study. (B) Experimental groups. (C) Prechallenge data points. ZIKV E-specific IgG titers are expressed as MFIs of the serum pools diluted 1:10 with normal BALB/c serum, simulating IgG titers expected *in vivo* after i.p. injection. Postchallenge data points are GMTs (with 95% CIs) from postchallenge BALB/c serum. (D) Neutralizing titers (PRNT_50_s) in sera from passively immunized mice 2 weeks postchallenge. The significance of differences between ZIKV PRNT_50_ values of the mouse groups receiving high-titer, low-titer, or naive SW mouse sera and the control group were determined by using a one-way ANOVA (Prism; GraphPad Software, Inc., San Diego, CA) and then by using Tukey’s multiple comparisons. (*, *P* < 0.05; **, *P* < 0.01.)

To evaluate whether antiserum from mice immunized with the ZIKV E vaccine formulation with CoVaccine HT or alum can provide passive protection, 100 μl of pooled sera with high or low antibody titers obtained from vaccinated SW mice (administered 10 μg ZIKV E with either CoVaccine HT or alum) (Fig. 2D) were transferred intraperitoneally (i.p.) to naive BALB/c mice one day before i.v. (tail vein) ZIKV challenge (Fig 5A and B). Antibody titers were measured 14 days after challenge to determine efficacy against productive virus infection. Both groups receiving either the low- or high-titer serum showed a decrease in anti-ZIKV E IgG of approximately 6- and 3-fold, respectively, compared to prechallenge titers. Due to the time point early after infection, mice receiving naive serum or no serum generated only low titers of ZIKV E-binding IgG antibodies (Fig. 5C); however, they showed consistent seroconversion. The relatively high MFIs observed from the high-titer serum transfer group postchallenge suggest residual IgG from the transfer remaining even 2 weeks after injection. Seroconversion is a sign of productive virus infection in unprotected animals, and therefore, the presence of anti-ZIKV NS1 titers postchallenge in all mice, including those receiving high-titer antisera (data not shown), suggests a low level of virus replication rather than sterilizing immunity; nevertheless, passive immunization appears to result in protection sufficient to prevent generation of significant antibody responses against the E protein, which otherwise indicate widespread virus dissemination.

The presence of neutralizing antibodies postchallenge (Fig. 5D) was determined in mice receiving high-titer, low-titer, or naive-mouse sera or in control mice (no serum transfer), and the data comparing the neutralizing antibody titers and the MIA titers for the pre- and postchallenge sera are shown in Table 1. Determination of prechallenge neutralizing antibody titers in individual mice was not possible due to standard Institutional Animal Care and Use Committee (IACUC)-imposed restrictions on the volume of blood drawn from live animals, which prevented sufficient volumes required for PRNT assays to be drawn. The difference in neutralizing titers in the transferred sera between the *de novo* high-titer (EC_50_ = 1,153) and low-titer (EC_50_ = 35) antisera was >30-fold. We believe that the relatively low level of neutralizing antibodies postchallenge in mice receiving high-titer antisera compared to levels in all other groups suggests that transfer of high-titer antisera provided passive protection by inhibition of virus replication, resulting in no primary antibody response in this group (Fig. 5D), consistent with no or very limited virus replication in these animals.

**TABLE 1:** Comparison of ZIKV IgG antibodies by MIA and neutralizing-antibody titers by PRNT in pre- and postchallenge sera from passively immunized mice

Characteristics of transferred serum	*De novo* prechallenge		Postchallenge	
	High titer	Low titer	High titer	Low titer
MIA MFI^a^	4,732^b^	219^b^	1,422^b^	84^b^
PRNT_50_	1,153^c^	35^c^	153^*d*^	713^d^

Ratio of MFI to PRNT_50_ titer	4.1	6.3	9.3	0.1

a ZIKV IgG antibodies were measured using the microsphere immunoassay (MIA) and are expressed as median fluorescence intensities (MFIs).

b Data are from Fig. 5C.

c PRNT_50_s from assays conducted on 1:10 dilutions (in normal BALB/c mouse serum) of serum pools that were used for passive transfer.

d Data are from Fig. 5D.

The simulated high-titer SW mouse serum pool (estimated by mixing SW mouse serum pools with naive BALB/c mouse sera at a 1:10 ratio) exhibited a PRNT_50_ of 1,153 (Table 1), while the anti-ZIKV E titer, measured by MIA, was 4,732 (Table 1). The simulation using this dilution is based on the proportion achieved by adding SW mouse sera (100 μl) to the total serum volume of a BALB/c mouse (~900 μl). In contrast, assays of the postchallenge sera from individual mice receiving the high-titer sera yielded a geometric mean titer (GMT) of PRNT_50_s of 153 (Fig. 5D), while the GMT of IgG titers (expressed as MFIs) was 1,422 (Table 1). Thus, the ratio of antigen-binding antibody to neutralizing antibody in the transferred serum, 4.1 (4,732/1,153), is half the value of 9.3 (1421/153) seen in the postchallenge sera from the same group, suggesting that neutralizing antibody activity after challenge declined much faster than the antigen-binding activity.

## DISCUSSION

The results reported herein describe the development of a ZIKV recombinant subunit vaccine produced in *Drosophila melanogaster* S2 cells, its immunogenicity in three mouse strains, and its protective efficacy in a viremia model. Stably transformed S2 cell lines yielded high quantities of truncated ZIKV E protein at a relatively high purity after single-step purification by IAC and is reactive with convalescent-phase sera. The crystal structures of soluble ZIKV and DENV E proteins expressed using this platform have been elucidated previously (31, 32) and appear to be “native-like”; however, these soluble proteins did not form virus-like particles (VLPs) due to the truncation of the membrane anchor. Upon administration of two doses of ZIKV E protein formulated with two clinically relevant adjuvants, maximum antibody titers were observed in mice 2 weeks after the second dose, which is consistent with the humoral response from other adjuvanted subunit vaccines (27). ZIKV E with CoVaccine HT elicited higher antibody titers than E protein with alum upon the initial dose, although antibody titers equilibrate after subsequent booster immunizations.

The immunogenicities of our ZIKV E vaccine candidates were compared using three different mouse strains with different immunological backgrounds. IgG titers for the vaccinated BALB/c (Th2-dominant) and C57BL/6 (Th1-dominant) mice were consistent and displayed no difference between the two adjuvants after the second dose. Thus, the vaccine candidate was found to be equally immunogenic in a strain with a humoral bias (BALB/c) or a cell-mediated bias (C57BL/6). However, a wide variance in IgG titers from immunized SW (outbred) mice was observed from sera collected after both the first and the second dose for both adjuvanted formulations. This could be attributable to a suboptimal antigen dose (5 versus 10 μg) and to the outbred status of this strain. For immunization of heterogeneous populations (e.g., humans), a dose-response study is warranted in order to optimize the immunizing dose for maximal seroconversion. Such a study could be conducted with NHPs prior to initiating a human clinical trial. High neutralizing antibody titers have been correlated with protection from flavivirus infections (33, 34). We have demonstrated that two doses of the ZIKV E vaccine candidate induce high levels of neutralizing antibodies in BALB/c and C57BL/6 mice and somewhat lower, but still highly significant, titers in SW mice. For strains administered the same vaccine formulation, no difference was observed among the vaccinated groups, indicating a favorable humoral response regardless of the Th1 or Th2 dominance of the mouse strain. While high antigen-binding IgG titers are not always correlated with induction of high neutralizing antibody levels, the ZIKV E vaccine candidate used in this study appears to stimulate both high antigen-binding and neutralizing antibody titers, suggesting that the recombinant subunit ZIKV E antigen is presented to the immune system in a proper conformation.

The protective efficacy of the recombinant subunit vaccine candidate was evaluated in a mouse model of systemic viremia. BALB/c mice were chosen for this model based on previous work by Larocca et al. (20), who showed robust ZIKV replication in these mice when they were inoculated intravenously. Results from ZIKV challenge with the PRVABC59 strain in immunized BALB/c mice show complete protection against viremia after a two-dose regimen, assessed by plaque assay and corroborated by a lack of anamnestic antibody responses postchallenge. Protection from high-level virus replication is inferred based on the lack of increased IgG titers postchallenge in mice receiving two doses of either of the adjuvanted formulations. The rapid increase in IgG titers after challenge in groups receiving one dose of ZIKV E with either adjuvant is consistent with limited virus replication in these animals and reactivation of memory B cells. A single dose of antigen with CoVaccine HT generated higher IgG titers than with alum. These observations support the protective efficacy of the vaccine candidate, especially the formulation containing CoVaccine HT, as it is able to consistently produce higher initial IgG titers than the formulation using alum and maintains high IgG levels after subsequent immunizations.

A passive-protection experiment showed that sufficiently high anti-ZIKV E IgG and PRNT_50_ titers alone protected individuals from virus dissemination. These results are consistent with the protection from the Brazil ZIKV2015 strain seen in BALB/c mice receiving an i.v. adoptive transfer of purified IgG from prM-E DNA vaccine-immunized mice and rhesus macaques (20). While the transfer of low ZIKV E-specific antibody titer serum to naive mice generated slightly lower PRNT_50_ titers (GMT = 713) after virus challenge than were seen in naive-mouse serum recipients (GMT = 934) or in the control group (GMT = 939), these differences were not statistically significant. The depletion of neutralizing antibodies postchallenge in the high-titer serum transfer group was likely the result of potent neutralization upon challenge, yielding virus-antibody complexes which are subsequently cleared by the host and thus not available for further virus neutralization. Nonneutralizing, but antigen-reactive, antibodies might persist and continue to be observed in the MIA, consistent with successful passive immunoprophylaxis. In contrast, recipients of low-titer sera showed a significant increase in PRNT_50_ titers coupled with a drastic decrease in the ratio of antigen-binding antibody to neutralizing antibody titers (from 6.3 to 0.1). This sign of seroconversion subsequent to virus challenge indicates an absence of protection and mirrored the response seen in recipients of the naïve-mouse serum pool and the control group (postchallenge ratios of 0.05 for both groups). Since the PRNT_50_ titer of the low-titer serum transfer group did not exceed the PRNT_50_ titers of groups not administered immunoprophylaxis, we conclude that the presence of high anti-ZIKV titers protects against viremia but that low titers may provide an incremental degree of protection, although not sufficient to prevent viremia.

Success with passive immunoprophylaxis by antiserum generated using the insect cell-expressed recombinant vaccine subunit antigen highlights the quality of the IgG response generated using ZIKV E as a lead antigen. Passive immunization with E-specific antibodies has also been shown to provide protection against other flavivirus diseases (35, 36). Treatment of BALB/c mice with E-specific antibodies provided protection from a lethal i.p. challenge against tick-borne encephalitis virus (TBEV) (37), similar to our observations, while increased NS1-, and lower E-specific antibody titers were observed postchallenge. Recovery of infectious TBEV after passive immunization and challenge of mice indicated a low level of virus replication, but nevertheless, protection against highly lethal disease was observed. Likewise, treatment with E-specific monoclonal antibodies reduced morbidity and increased survival against intracerebral (i.c.) challenge with JEV, WNV, and DENV-2 (35, 36, 38) in SW mice. Clearly, the presence of E-specific antibodies is positively associated with flavivirus disease protection.

While antigen-specific antibodies protect against homoserotypic infections, progression to severe disease is a concern with preexisting flavivirus immunity to a different serotype or different flavivirus. Although high virus-neutralizing titers are associated with protection, generation of antibody with suboptimal neutralizing levels may promote antibody-dependent enhancement (ADE) observed in other flaviviruses, such as DENV, which can complicate vaccine safety in areas where cocirculation is endemic. Several studies have shown that ZIKV-induced antibodies have the ability to enhance DENV and other flavivirus infection *in vitro* (39–41). Conversely, many more studies have documented the reverse, namely, DENV sero-cross-reactive antibodies driving ADE of ZIKV infection both *in vitro* (41–44) and in murine models (41, 45), while other studies have shown similar enhancement with WNV convalescent-phase serum (45–47). Yet despite the mounting *in vitro* evidence, preexisting flavivirus immunity appears not to cause severe ZIKV disease in rhesus macaques (48).

A contemporary study examined the immunogenicity of E protein domain III (EDIII) as a protein subunit vaccine candidate purified from transformed *Escherichia coli* inclusion bodies (49). This vaccine candidate induced high IgG1 antibody titers as well as ZIKV-neutralizing antibodies. Sera from immunized C57BL/6 mice showed no indication of eliciting ADE, which is notable considering that EDIII is known to contain many neutralizing antibody epitopes (49). Whether recombinant ZIKV E protein alone can promote heterologous virus enhancement remains to be investigated, although another study of a ZIKV VLP vaccine candidate expressing a full-length ZIKV E protein has shown no indication of enhancement from immunized mouse sera in U937 cells (50), a finding that further validates *in vivo* observations using the recombinant ZIKV E subunit vaccine candidate presented here.

Recombinant subunit vaccines have an excellent safety profile, as the immunogen is nonreplicative and there are no concerns regarding prior vaccine vector immunity. These advantages are especially beneficial considering stringent precautions required for the demographic most severely affected by this flavivirus infection (e.g., pregnant women and the elderly). Previous candidate recombinant subunit vaccines have been developed for WNV, DENV serotypes 1 to 4, Chikungunya virus (CHKV), and Ebola Virus (EBOV) and have been shown to be protective in mice (25, 27, 51, 52) and in NHPs (28, 53). WNV and DENV vaccine candidates based on the same platform have been evaluated in phase 1 clinical trials and have been shown to be well tolerated and immunogenic (54, 55).

In summary, we present an immunogenic recombinant ZIKV subunit vaccine candidate containing the E protein adjuvanted with either alum or CoVaccine HT. Two immunizations of the vaccine induced a robust antigen binding IgG titer and high levels of neutralizing antibodies in three different mouse strains. Furthermore, twice-immunized mice did not develop viremia after live-virus infection and did not mount an anamnestic antibody response after virus challenge, suggesting that immunization was sufficient to prevent virus replication. Passive transfer of immune mouse sera to naive mice corroborated the results seen in the virus challenge study of actively immunized mice and demonstrated the importance of the humoral response in protection. Based on the results reported herein, further development of the ZIKV E protein vaccine candidate is warranted.

## MATERIALS AND METHODS

### Ethical statement.

Mouse experiments were approved by the University of Hawai’i Institutional Animal Care and Use Committee (IACUC) and conducted in strict accordance with local, state, federal, and institutional policies established by the National Institutes of Health and the University of Hawai‘i IACUC. The University of Hawai’i’s John A. Burns School of Medicine (JABSOM) Laboratory Animal Facility is accredited by the American Association for Accreditation of Laboratory Animal Care (AAALAC). All animal experiments were conducted in consultation with veterinary and animal care staff at the University of Hawai’i. Mice were bred in colonies at JABSOM from original stocks obtained from Taconic Biosciences Inc. (Hudson, NY) (SW, BALB/c) or the Jackson Laboratory (Bar Harbor, ME) (C57/BL6).

### Expression and purification of ZIKV E and NS1 proteins.

Expression vectors (based on pMT-BiP [Invitrogen Corporation, Carlsbad, CA]) were generated using synthetic genes optimized for expression in insect cells generated based on (i) the entire ZIKV prM protein followed by amino acids 1 to 408 of ZIKV E or (ii) full-length NS1 based on the French Polynesian strain (GenBank accession number KJ776791). ExCell420 medium (Sigma-Aldrich, St. Louis, MO)-adapted *Drosophila* S2 cells were cotransfected with the expression vector and a selectable marker plasmid, pCoHygro, using the Lipofectamine LTX with Plus reagent (Invitrogen Corporation, Carlsbad, CA) by following the manufacturer’s instructions. Stably transformed cell lines were created by selection with medium containing added hygromycin B to a final concentration of 300 μg/ml. To verify expression, transformants were induced with culture medium containing 200 μM CuSO_4_, followed by analysis on SDS-PAGE and Western blotting (25). (While not part of the vaccine candidate, NS1 was also expressed and purified in order to detect antibodies to NS1 in mice after viral challenge to confirm successful infection.)

Antigens were produced in a WAVE bioreactor (GE Healthcare, Piscataway, NJ) using 2- or 10-liter bag sizes (and 1- to 5-liter culture volumes) and were subsequently purified by IAC. The mouse monoclonal antibody 4G2 was coupled to *N*-hydroxysuccinimide (NHS)-Sepharose (GE Healthcare, Piscataway, NJ) at 10 mg/ml. For ZIKV E purification, S2 cell culture medium containing recombinant protein was clarified by centrifugation and sterile filtered (0.2-µm pore size). The material was then loaded onto the IAC column at a linear flow rate of approximately 1 ml/min. After the supernatant was loaded, the matrix was washed with 10 mM phosphate-buffered saline (PBS), pH 7.4, containing 0.05% (vol/vol) Tween 20 (PBST, 140 mM NaCl), followed by a wash with PBS, pH 7.4. Bound protein was eluted from the IAC column with 20 mM glycine buffer, pH 2.5. The eluent was neutralized with 1 M phosphate buffer, pH 7.4, and the buffer was exchanged with PBS and concentrated using Centricon Plus-20 devices (EMD Millipore, Burlington, MA).

Similar procedures were followed for polyhistidine-tagged NS1 purification using a HisTrap FF column (GE Healthcare, Piscataway, NJ) per the manufacturer’s instructions. The recombinant protein was loaded at a linear flow rate of approximately 1 ml/min and washed with PBS containing 300 mM NaCl, pH 7.4. Bound protein was eluted on a linear gradient of up to 0.5 M imidazole in the same buffer, and then the buffer was exchanged with PBS and concentrated as described above.

The purified products were analyzed by SDS-PAGE by Coomassie blue staining followed by Western blotting and quantified by UV absorption. Purified recombinant proteins were filter-sterilized and stored at −80°C. A control “null” antigen was prepared by buffer exchange and concentrating supernatants from untransformed S2 cells grown under conditions identical to those used for the S2 cell lines expressing recombinant proteins and was used as a control to represent potential insect cell-derived contaminants (refer to “Luminex-based MIA” below).

### Western blotting.

The supernatant of an S2 cell culture collected one week postinduction was mixed with NuPAGE lithium dodecyl sulfate sample buffer (Thermo Fisher, Waltham, MA), boiled for 10 min, and run on NuPAGE 4 to 12% bis-Tris protein gels (Thermo Fisher, Waltham, MA). Protein was transferred to a nitrocellulose membrane with a Bolt miniblot module (Thermo Fisher, Waltham, MA) and blocked with 1% nonfat dry milk (Nestlé United States Inc., Solon, OH) in PBST, pH 7.4, with 0.01% sodium azide for 1 h at room temperature or overnight at 4°C. Membranes were probed with a 1:3,000 dilution of flavivirus E-specific 4G2 antibody (1.6 mg/ml) or convalescent-phase PRVABC59 ZIKV-infected mouse or NHP serum at a dilution of 1:1,000 for 1 h at room temperature. After being washed with PBST, membranes were incubated with alkaline phosphatase-conjugated, goat anti-mouse or anti-human IgG antibody (Southern Biotech, Birmingham, AL) for another hour. The membranes were washed three times with PBST and developed with a nitroblue tetrazolium (NBT)-BCIP (5-bromo-4-chloro-3-indolylphosphate) (Promega, Madison, WI) solid-phase alkaline phosphatase substrate solution.

### Coupling of microspheres with recombinant ZIKV-E and NS1 antigens.

The coupling of microspheres with ZIKV E or NS1 proteins was performed as described previously (56, 57). Internally dyed, carboxylated, magnetic microspheres (MagPlex-C) were obtained from Luminex Corporation (Austin, TX, USA). A two-step carbodiimide process recommended by Luminex was used to link 10 µg of purified ZIKV E or NS1 to the surfaces of 1.25 × 10^6^ microspheres. The antigen-conjugated microspheres were stored in 250 µl of PBN buffer (PBS with 1% bovine serum albumin fraction V [OmniPur] and 0.05% Ultra sodium azide [Sigma-Aldrich, St. Louis, MO]) at 4°C. Microspheres dyed with spectrally different fluorophores were also coupled with the untransformed S2 cell culture supernatant (null), bovine serum albumin, and PBS for controls.

### Luminex-based MIA.

Microspheres coupled with ZIKV-E or NS1, the untransformed S2 supernatant, bovine serum albumin (BSA), and PBS were pooled in PBS-1% BSA (PBS-BSA) at a dilution of 1:200. Fifty microliters of the coupled microsphere immunoassay (MIA) suspension was added to each well of black-sided 96-well plates. Serum samples were diluted 1:100 in PBS-BSA, and 50 µl was added to the microspheres in duplicate and incubated for 30 min on a plate shaker set at 700 rpm in the dark at room temperature. The plates were then washed twice with 200 µl of PBS-BSA using a magnetic plate separator (Millipore Corp., Billerica, MA). Fifty microliters of red phycoerythrin (R-PE)-conjugated *F*(ab′)2 fragment goat anti-mouse IgG specific to the Fcγ fragment (Jackson ImmunoResearch, Inc., West Grove, PA) was added at 1 µg/ml to the wells and incubated another 45 min. The plates were washed twice, as described above, and microspheres were then resuspended in 100 µl of sheath fluid and analyzed on a Luminex 100 apparatus (Luminex Corporation, Austin, TX). Data acquisition detecting the MFI was set to 50 beads per spectral region. Antigen-coupled beads were recognized and quantified based on their spectral signature and signal intensity, respectively.

Cutoff values were calculated as the average MFI of 25 serum samples from mock-treated BALB/c mice plus three standard deviations (Microsoft Office Excel). Serum samples with MFI values greater than the cutoff were considered positive. Control beads were coupled with BSA and the S2 cell supernatant using the aforementioned coupling protocol (56) to check for nonspecific attachment of serum proteins to the microspheres. The signals generated with BSA-, S2 supernatant-, and PBS-coupled beads were below the cutoff.

To establish the correlation between MFI and antibody titer, single-point (1:100 dilution) MFI values from seven serum samples containing different anti-ZIKV E titers were plotted against their EC_50_s. EC_50_ titers were determined by MIA on mouse antisera derived by using six 4-fold serial dilutions of each serum sample, starting at 1:25, and measuring the MFI at each dilution. A sigmoidal dose-response computer model (Prism, GraphPad Software, Inc., San Diego, CA) was used to generate the values for the EC_50_s from these data.

### Virus stock and cell culture.

Vero cells were grown in Hanks balanced salt medium 199 (M199), containing 2 mM l-glutamine, 5% fetal bovine serum (FBS), and 100 U/ml penicillin-streptomycin (Invitrogen, Carlsbad, CA), maintained at 37°C, and humidified in 5% CO_2_. ZIKV Puerto Rican strain PRVABC59 was obtained from the American Type Culture Collection (ATCC; Manassas, VA) and amplified in Vero cells. Virus titers were determined by plaque assay as described previously (59).

### Vaccination, serum collection, and challenge.

All mice (ages at the first immunization ranged from 6 to 9, 6 to 10, and 15 to 18 weeks for BALB/c, C57/BL6, and SW mice, respectively) were immunized intramuscularly (i.m.) with 5 to 10 μg of ZIKV E protein (specified above) with or without adjuvants using an insulin syringe with a 29-gauge needle. The adjuvants used were alum (2% Alhydrogel adjuvant [InvivoGen, San Diego, CA] or Imject alum [Thermo Fisher, Waltham, MA]) or CoVaccine HT (Protherics Medicines Development Ltd, London, United Kingdom), a squalane-in-water emulsion containing sucrose fatty acid sulfate esters immobilized on submicron-sized oil droplets (60). Sera were collected 2 weeks postimmunization by tail bleeding or cardiac puncture for terminal bleeds. ZIKV E-specific IgG antibody titers in mouse sera were measured using the MIA as described above. Challenge with 100 PFU of the Puerto Rican ZIKV strain PRVABC59 was conducted via injection of 100 µl of diluted virus stock into the tail veins of mice 11 to 13 weeks old at the time of challenge. To determine the kinetics of viremia, blood was collected daily for 5 days postchallenge and a plaque assay of serum samples was performed as described previously (61).

### PRNT.

Sera from individual animals were heat inactivated by incubation at 56°C for 30 min. The titer of anti-ZIKV neutralizing antibody was measured in the serum using the plaque reduction neutralization test (PRNT) as described previously (59). Briefly, a series of 4-fold dilutions, starting at 1:20, of each sample was prepared using M199 medium, and the samples were incubated overnight with medium containing previously titrated virus at 4°C in a 1:1 (vol/vol) ratio to generate approximately 50 PFU of virus per well. The antibody-virus complex was then added to Vero cells in duplicate and incubated at 37°C for 1 h. Cells were overlaid with M199 containing 1% agarose and incubated for 72 h. A second overlay of 1% agarose in M199 containing 1.5% neutral red (Sigma, St. Louis, MO) was added, and plaques were counted 24 to 48 h later. PRNT_50_ values, the serum dilutions yielding 50% virus neutralization, were generated using a fixed, or variable-slope sigmoidal dose-response computer model (Prism; GraphPad Software, Inc., San Diego, CA).

### Passive-transfer studies with BALB/c mice.

Sera from SW mice immunized with ZIKV E with adjuvant were pooled and filter sterilized, and 100 μl of undiluted serum was injected i.p. into 11-week-old BALB/c mice 1 day prior to virus challenge. Serum collection by tail vein bleeding and cardiac puncture was performed 2 and 3 weeks after challenge, respectively. ZIKV E-specific IgG antibody titers in mouse sera were measured by MIA as described above. The presence of neutralizing antibodies was determined by PRNT.

### Statistical analysis.

Determination of significant differences in IgG antibody titers between groups of mice was performed using an unpaired *t* test. Significant differences in ZIKV PRNT_50_ values among sera from passively protected BALB/c mice (Fig. 5D) receiving high-titer, low-titer, or naive SW mouse serum or from the control mouse group were determined using a one-way analysis of variance (ANOVA), followed by Tukey’s multiple-comparison test. The Fisher exact probability test was used to determine significance between the numbers of viremic mice in vaccinated or control (nonvaccinated) groups subsequently challenged with live virus. All statistical analyses were performed with a commercial statistical program (Prism; GraphPad Software, Inc., San Diego, CA). A *P* of <0.05 was considered significant in one-tailed tests comparing vaccinated and control groups and comparing responses to primary vaccination and responses to each subsequent vaccination and in two-tailed tests comparing different vaccinated groups (GraphPad Prism).
